# Rapidly Growing Giant Squamous Cell Carcinoma of the Head: A Case Report

**DOI:** 10.7759/cureus.59630

**Published:** 2024-05-04

**Authors:** Zachary Leal, Mikayla Hobbs, Razia Gill, Megan Banfield, Mustapha Akhdar, Damian Casadesus

**Affiliations:** 1 Internal Medicine, Jackson Memorial Hospital, Miami, USA; 2 Medicine and Surgery, Jackson Memorial Hospital, Miami, USA

**Keywords:** head and neck squamous cell carcinoma (hnscc), giant squamous cell carcinoma, cutaneous oncology, ­skin cancer, squamous cell carcinoma (scc)

## Abstract

Giant squamous cell carcinoma (GSCC) of the skin arising on the head presents a distinctive clinical challenge due to its rarity, aggressive behavior, and potential for disfigurement. A male in his 70s with a history of tobacco cigarette use presented to the emergency department with a painful, bleeding mass on the right parietal scalp. On admission, a brain CT revealed a fungating mass with no cortical breakthrough or osseous erosion, measuring 7.9 x 5.7 x 2.5 cm. An ultrasound-guided tissue biopsy was performed and revealed poorly differentiated squamous cell carcinoma. The patient was discharged home with instructions from oncology to continue with outpatient treatment. At this time, the prognosis is good if treatment is received.

## Introduction

The incidence of cutaneous squamous cell carcinoma (cSCC) has steadily been increasing and now accounts for 20% of nonmelanoma skin cancer (NMSC), making it the second most common skin malignancy in the United States [1]. When caught early, and treated appropriately, a majority of cSCC cases can be treated successfully. However, when neglected, severe disfigurement and growth may occur [2]. cSCC lesions have a broad clinical presentation. It may appear as papules, plaques, nodules, smooth in texture, scaly, ulcerated, pruritic, tender, or even asymptomatic [3]. Patients may also report neuropathic symptoms such as numbness, paresthesia, burning sensations, or paralysis which suggests perineural invasion of the tumor and a poorer prognosis [3]. Once cSCC tumors extend beyond 5 cm in diameter, they are classified as giant squamous cell carcinomas (GSCC) and become more complicated to treat, as well as more likely to present with metastasis or recurrence [2].

The unique anatomical considerations of the head region contribute to the complexities of managing these tumors, as their proximity to vital structures poses a risk of functional impairment and aesthetic compromise. The diagnosis of GSCC on the head relies on a combination of clinical assessment and histopathological confirmation. Given the potential for mimicking benign lesions or other skin conditions, a thorough biopsy is essential. Along with the diagnosis of SCC, it is crucial to stage the lesion and determine the extent of cancer spread. Multiple imaging modalities are used to determine such important aspects of the cancer. Lymph node biopsies are used to evaluate potential metastasis, while CT and MRI scans are useful for assessing the spread and involvement of distant organs. The main therapeutic approach for this variant of squamous cell carcinoma (SCC) is surgical excision with radiation often employed as an adjuvant therapy. The prognosis of this variant of cancer may vary, and it is important to involve various medical specialties to tailor a collaborative and individualized treatment plan. We present a patient with a rapidly growing, highly vascularized, and poorly differentiated GSCC of the head.

## Case presentation

A male in his 70s with a past medical history of coronary artery disease with STEMI status post percutaneous coronary intervention, hypertension, and tobacco cigarette use presented to the emergency department (ED) due to unbearable pain associated with a mass on the right side of his scalp. He first noticed the mass in the mirror two months prior, which at the time was painless and the size of a quarter. The mass continued to rapidly grow, eventually becoming painful, and started bleeding about one month before the ED visit. It had continued to bleed every day since then. The patient stated the pain progressed over time and described his current pain as constant with a rating of 17/10. He denied fever, ear pain, vision changes, eye pain, and throat pain. He was seen by his primary care physician for the mass and was given several courses of antibiotics and analgesics, which did not result in improvement. He was scheduled to see a dermatologist in the next month, but the pain became unbearable causing him to seek care earlier. The patient is a tobacco smoker and reports drinking 1-2 beers per week. The only medication the patient reported taking at home was Pepcid. 

**Figure 1:**
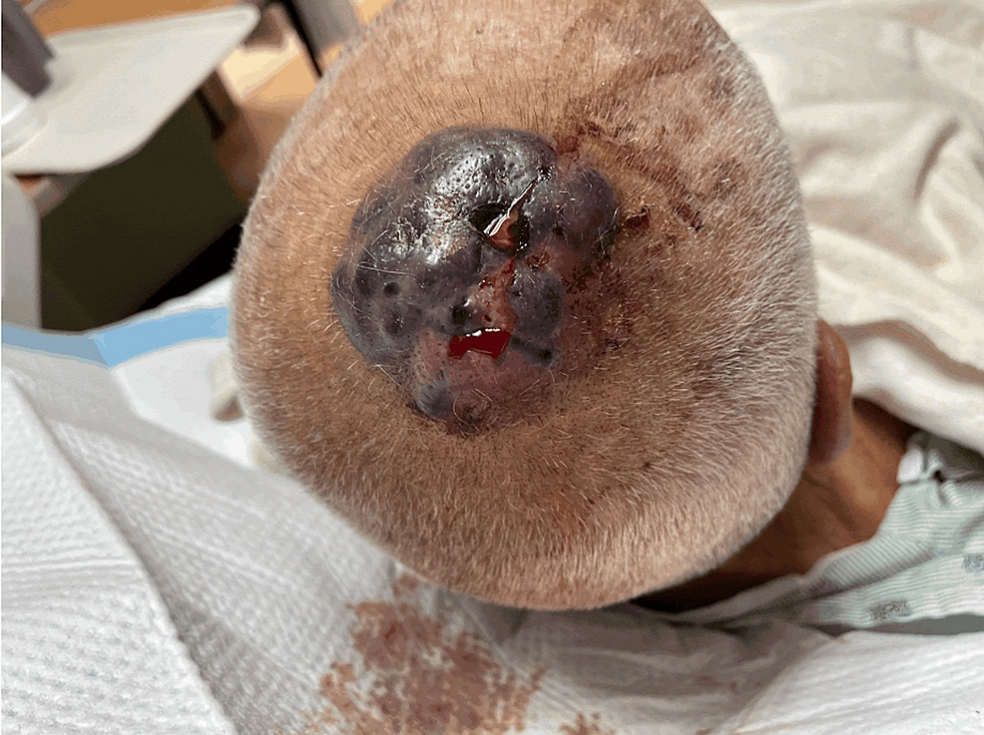
Fungating violaceous mass (6 × 5 cm) with serosanguineous fluid on the right parietal scalp.

On physical exam, vitals were stable with a blood pressure of 125/61 mmHg, a temperature of 36.9°C, a heart rate of 64 beats per minute, a respiratory rate of 20 breaths per minute, and an oxygen saturation of 98% on room air. The patient appeared in distress due to pain. The examination of the head showed a tender 6 × 5 cm fungating violaceous mass draining serosanguineous fluid on the right parietal scalp (Figure 1). The rest of the physical examination was unremarkable.

**Figure 2:**
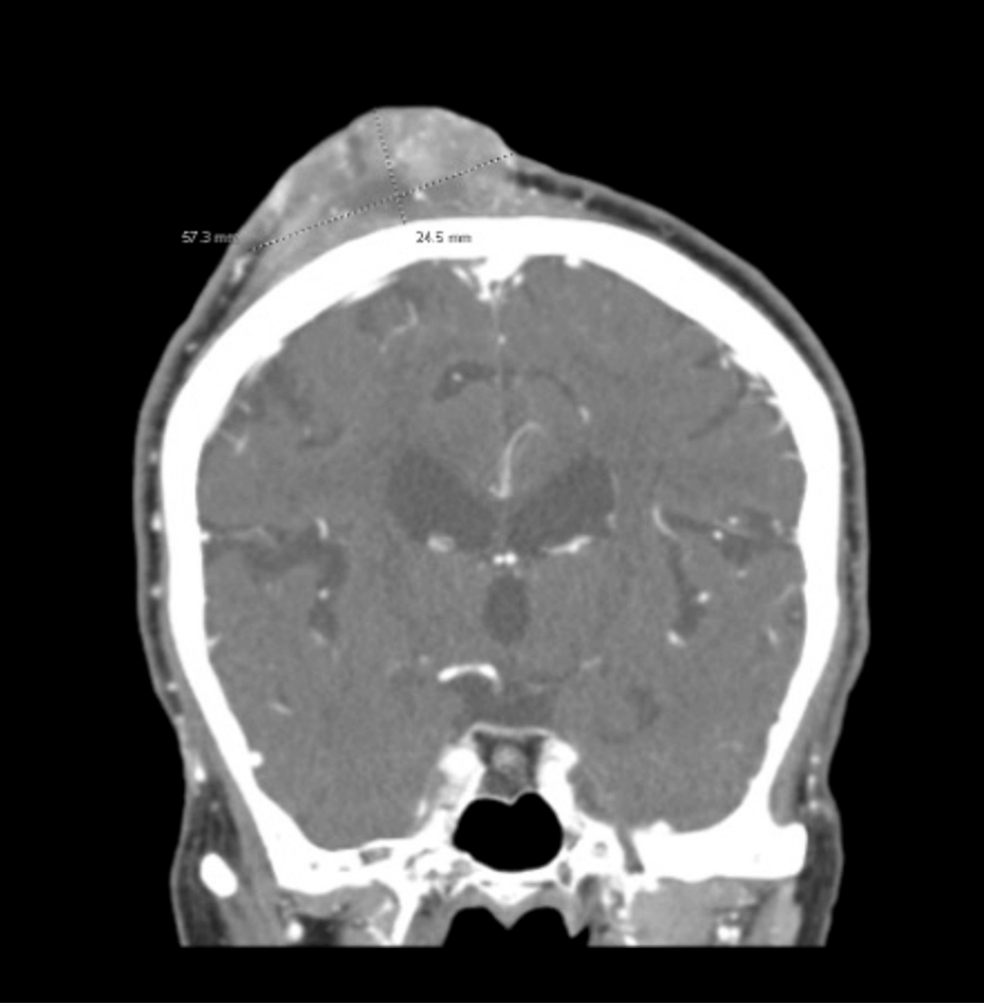
CT of the head with contrast showing mass overlying the right parietal scalp.

**Figure 3:**
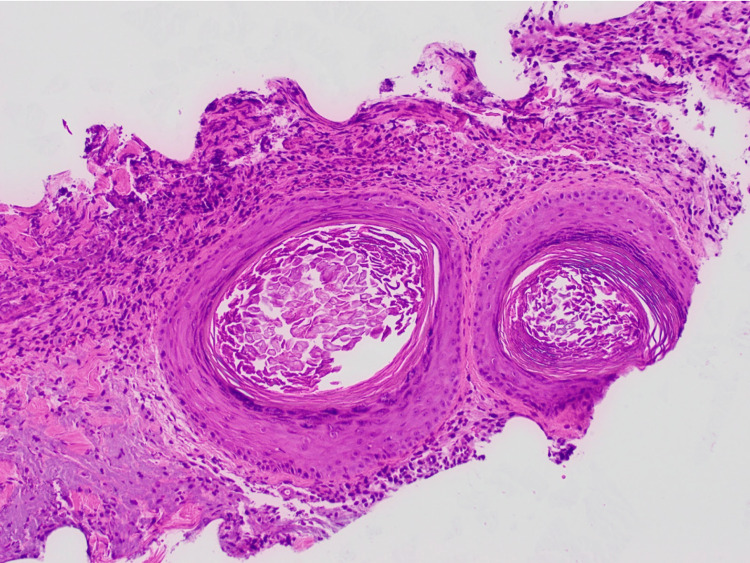
Histopathological study of scalp mass showing poorly differentiated squamous cell carcinoma.

Relevant laboratory results on admission were remarkable for normocytic anemia, critical hypokalemia, and lactic acidosis (Table 1). Differential diagnoses included a collection of fluid, osteomyelitis, keratoacanthoma, sebaceous carcinoma, SCC, and other malignancies. The patient was admitted to the hospital and started treatment with pain management and correction of potassium levels. A CT of the head revealed a 7.9 x 5.7 x 2.5 cm heterogeneously enhancing, fungating mass overlying the right parietal scalp, with no cortical breakthrough, no osseous erosion, no enhancing masses of the cerebral parenchyma, and no mass effect or midline shift (Figure 2). An ultrasound-guided tissue biopsy was performed, and the patient was discharged home with the primary doctor and dermatology follow-up. The histopathological study revealed a poorly differentiated SCC (Figure 3). The patient returned to the hospital a month later because of increased pain. After treatment, the patient was scheduled for oncological treatment; however, the patient was lost to follow-up.

**Table 1:** Relevant laboratory values on admission with reference values.

Laboratory test	Patient laboratory value	Normal laboratory range
White blood cells	10,600/mm^3^	4500-11,000/mm^3^
Hemoglobin	12.3 g/dL	13.5-17.5 g/dL
Hematocrit	39%	41-53%
Platelets	179,000/mm^3^	150,000-400,000/mm^3^
Potassium	2.7 mEq/L	3.5-5.0 mEq/L
Prothrombin time	12.9 seconds	11-15 seconds
Partial thromboplastin time	32 seconds	25-40 seconds
International normalized ratio	0.96	<1.1
Red blood cells	3.8 million/mm^3^	4.3-5.9 million/mm^3^
Mean corpuscular volume	95.3 mm^3^	80-100 mm^3^
Lactic acid	2.5 mmol/L	<2 mmol/L

## Discussion

SCC is most common in white populations that have a fair complexion, blue eyes, and blonde/red hair, with most patients being white males in their 60s [1]. Factors that predispose patients to develop cSCC include light skin, exposure to sunlight, ultraviolet radiation, immunosuppression, human papillomavirus, chronic conditions that lead to scarring, genetic syndromes, and certain environmental exposures [4]. The main high-risk prognostic markers for cSCC include the tumor size, site, history of radiotherapy, immunosuppression, and perineural invasion [5].

GSCC is defined by its substantial size, often exceeding 5 cm or even 10 cm in diameter. Grossly GSCC often manifests as a large, ulcerated, exophytic mass with irregular borders and hemorrhage or weeping of the tumor mass frequently observed [6]. Histologically, GSCC exhibits features typical of conventional cSCC demonstrating marked cellular atypia, pleomorphism, and high mitotic activity, with keratin pearls and intercellular bridges [7]. GSCC is also characterized by its disproportionately large size and rapid growth rate, often leading to extensive tissue necrosis and inflammatory infiltrates.

The main feature that makes our patient rather notable is the remarkably rapid rate of tumor growth he experienced. Although a great amount of literature has been published discussing SCC, there remains to be limited literature that specifically discusses tumor growth rate and what affects it. Several hypotheses include states of immunosuppression, increased age, variations in gene expression signatures, unhealed prior cutaneous lesions, and smoking. Billingsley et al. hypothesized that tumor growth rate may be influenced by states of immunosuppression and may be due to a viral etiology [8]. Our patient, however, was not immunosuppressed. Another study found the rate of tumor progression to grow exponentially with increasing age, with an asymptote at the eighth decade of life [9]. This study may perhaps be a good explanation of our patient's rapid growth rate, as our patient was in fact in his eighth decade of life and, according to the study, indicates that patients at his age would experience the fastest rate of tumor growth compared to other age groups. Another study concluded that the use of gene expression profile testing such as DecisionDX-SCC demonstrated a high degree of analytical precision in risk classification after analyzing gene expression of primary tumor tissue, indicating that variations in genetic mutations may alter tumor progression and prognosis [10]. Another common feature seen in much of the published literature regarding rapidly growing cSCC is that the tumors in those patients may have progressed from prior cutaneous lesions, which did not appear to be the case in our patient. One study discussed a patient who developed aggressive cSCC and had a history of recurrent unhealed ulcerative scalp lesions [11]. Another risk factor in our patient that possibly impacted his tumor progression is his history of smoking, although it is unclear to what degree. There are numerous reports linking smoking to cSCC; however, one study found it had little to no association with development on the scalp [12].

## Conclusions

GSCC has the potential to be detected earlier with surveillance, follow-up, and early treatment. This tumor is known to have a great prognosis; however, the prognosis can be dismal if left untreated. Optimal treatment of GSCC consists of wide local excision with tumor-free margins, Mohs micrographic surgery, chemotherapy, radiation therapy, or a combination of the four. Physicians should be aware that an unresolved mass or lesion needs a timely biopsy for correct diagnosis to improve the survival of the patient. In our patient, constrained access to healthcare and a lack of comprehension regarding his medical condition contributed to a delayed diagnosis, potentially compromising the continuity of care and, consequently, leading to a potentially more unfavorable prognosis.
